# ICAM-1-Targeted Liposomes Loaded with Liver X Receptor Agonists Suppress PDGF-Induced Proliferation of Vascular Smooth Muscle Cells

**DOI:** 10.1186/s11671-017-2097-6

**Published:** 2017-05-03

**Authors:** Xu Huang, Meng-Qi Xu, Wei Zhang, Sai Ma, Weisheng Guo, Yabin Wang, Yan Zhang, Tiantian Gou, Yundai Chen, Xing-Jie Liang, Feng Cao

**Affiliations:** 1 Department of Cardiology, State Key Laboratory of Kidney Diseases, Chinese PLA General Hospital, Beijing, 100853 China; 2 Laboratory of Controllable Nanopharmaceuticals, CAS Key Laboratory for Biomedical Effects of Nanomaterials and Nanosafety, National Center for Nanoscience and Technology, Beijing, 100190 China

**Keywords:** Vascular smooth muscle cells, Liver X receptor, ICAM-1, Liposome

## Abstract

The proliferation of vascular smooth muscle cells (VSMCs) is one of the key events during the progress of atherosclerosis. The activated liver X receptor (LXR) signalling pathway is demonstrated to inhibit platelet-derived growth factor BB (PDGF-BB)-induced VSMC proliferation. Notably, following PDGF-BB stimulation, the expression of intercellular adhesion molecule-1 (ICAM-1) by VSMCs increases significantly. In this study, anti-ICAM-1 antibody-conjugated liposomes were fabricated for targeted delivery of a water-insoluble LXR agonist (T0901317) to inhibit VSMC proliferation. The liposomes were prepared by filming-rehydration method with uniform size distribution and considerable drug entrapment efficiency. The targeting effect of the anti-ICAM-T0901317 liposomes was evaluated by confocal laser scanning microscope (CLSM) and flow cytometry. Anti-ICAM-T0901317 liposomes showed significantly higher inhibition effect of VSMC proliferation than free T0901317 by CCk8 proliferation assays and BrdU staining. Western blot assay further confirmed that anti-ICAM-T0901317 liposomes inhibited retinoblastoma (Rb) phosphorylation and MCM6 expression. In conclusion, this study identified anti-ICAM-T0901317 liposomes as a promising nanotherapeutic approach to overcome VSMC proliferation during atherosclerosis progression.

**Electronic supplementary material:**

The online version of this article (doi:10.1186/s11671-017-2097-6) contains supplementary material, which is available to authorized users.

## Background

Studies in pathophysiology have shown that the activation, migration, and proliferation of vascular smooth muscle cells (VSMCs) play crucial roles in atherosclerosis. Notably, atherosclerosis is the primary cause of cardiovascular diseases and mortality in the industrialised nations [1, 2]. In general, VSMCs are quiescent and non-proliferative [3]; however, proliferation would be activated following vascular injury, which contributes to the pathogenesis of early atherosclerosis [4, 5]. Atherosclerotic lesions exhibit multiple molecular responses, including hyperglycemia, hypertension, and modified low-density lipoprotein expression, which damage endothelial cells and bind leukocytes and monocytes, causing lipoproteins to infiltrate the vascular intima [6]. Subsequently, macrophages engulf the oxidised low-density lipoproteins (LDL) and become foam cells [7]. As the inflammatory process continues, growth factors and cytokines, including the platelet-derived growth factors (PDGFs), promote VSMC migration from the medium to the intima and transition from a quiescent contractile state to an active synthetic state. This transition is the basis for atherosclerosis and restenosis [8].

Multiple approaches have attempted to weaken VSMC activation and proliferation. It has been demonstrated that LXR (LXRα and LXRβ) activation in macrophages and VSMCs regulated cholesterol homeostasis and inhibited mitogen-induced VSMC proliferation [9, 10]. LXRs (LXRα and LXRβ) are members of the nuclear hormone receptor family of transcription factors that inhibit VSMC proliferation and G_1_ → S phase progression by (i) preventing phosphorylation of retinoblastoma (Rb) protein at Ser807/811 and (ii) inhibiting the expression of minichromosome maintenance protein 6 (MCM6). Phosphorylation of Rb promotes cell cycle progression by releasing transcription factors, the S phase transcription factor E2F, that could induce gene expression required for DNA synthesis. The MCM6 is the machinery downstream of Rb phosphorylation that regulates the DNA replicative. Therefore, LXR agonists show great potential to inhibit VSMC proliferation. However, there hydrophobicity is the major barrier to their clinical use [10, 11].

Knowledge of atherosclerosis pathogenesis has provided opportunities for creative prevention strategies and treatments, including the use of nanotherapeutic approaches [12]. The application of nanotechnology in medicine has provided credible strategies, including multimodal imaging and nanomedicine with sustained releases [13–15]. Phosphatidylcholine (PC) nanoparticles exhibit targeting ability for MRI and imaging of atherosclerotic plaques in mice [16]. Nanoparticle-based drug delivery system (NDDs) is a promising therapeutic vector to improve the therapy efficiency for atherosclerosis treatment. NDDs overcomes many of the shortcomings that conventional therapies suffer from, including low water solubility and the inability to deliver drugs across a range of biological barriers [17]. As far as we know, few reports on the targeted therapeutics based on NDDs have been presented to inhibit VSMC activation and proliferation. It has been found that after PDGF stimulation, the expression of intracellular adhesion molecule-1 (ICAM-1) by VSMCs was greatly upregulated, which binds multiple types of inflammatory cells [18]. So, ICAM-1 could be identified as a marker for targeted drug delivery.

**Fig. 1:**
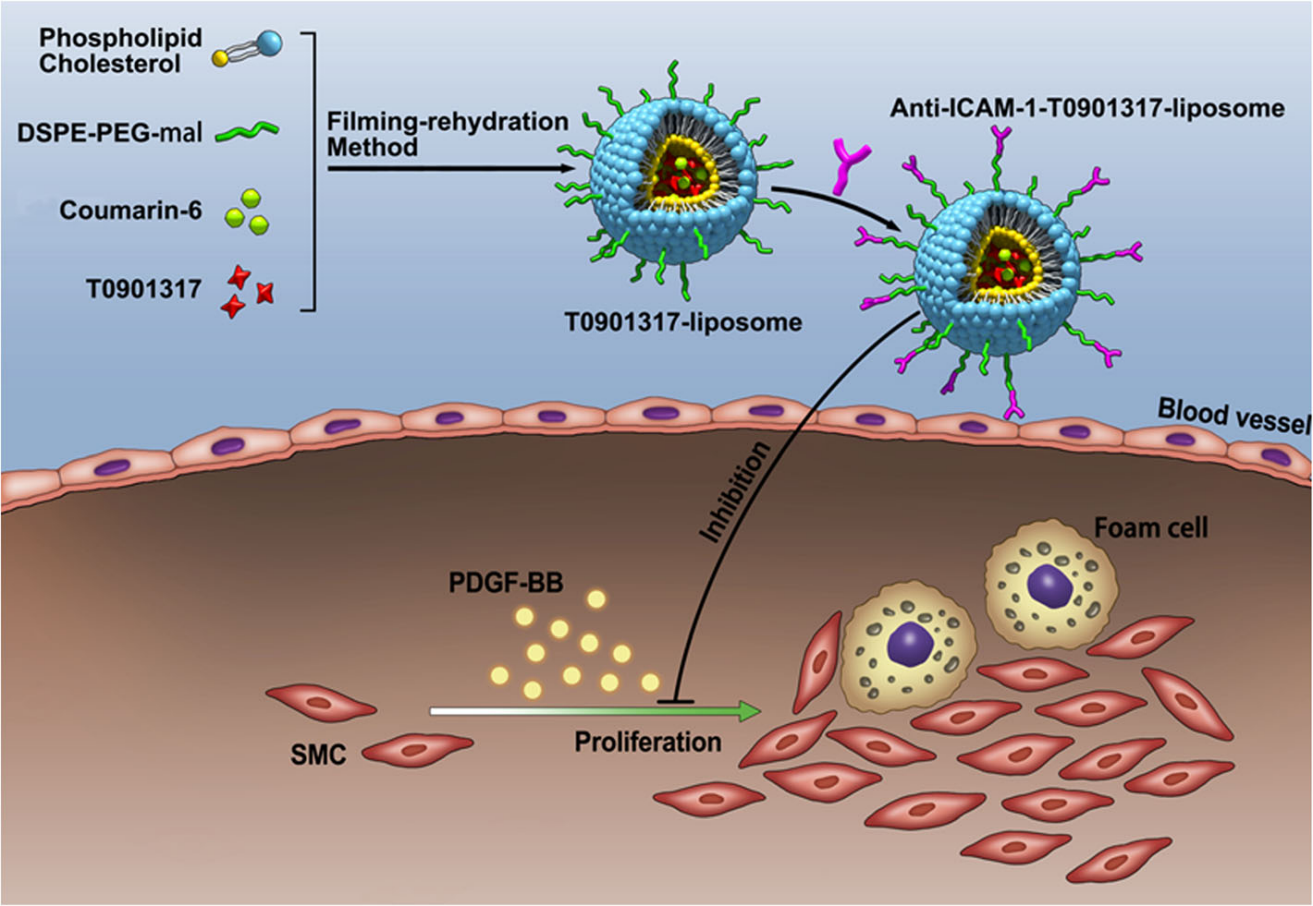
The scheme representing the subject of the article shows that the anti-ICAM-1-T0901317 liposomes were prepared by filming-rehydration method. Some specific materials contain phospholipid, cholesterol, T0901317, DSPE-PEG-mal, coumarin-6, and anti-ICAM-1. And the anti-ICAM-1-T0901317 liposomes can inhibit PDGF-induced proliferation of vascular smooth muscle cells effectively

Based on these considerations, herein, we developed a nanotherapeutic platform based on liposomes for targeted delivery of LXR agonist, T0901317, to inhibit the proliferation of VSMCs. ICAM-1 antibodies were conjugated to the liposomes to improve the therapeutic efficiency. The schematic presentation of the as-prepared anti-ICAM-1-T0901317 liposomes is shown in Fig. 1a, and the nanoparticles were fully characterised. Subsequently, the cellular uptake of the liposomes was investigated by CLSM and flow cytometry. The antiproliferation efficiency and the underlying mechanism were evaluated by CCK8 assay, BrdU staining, cell cycle assay, and western blotting.

## Methods

### Materials

PC from eggs, cholesterol (Chol), PEGylated (Mw¼2000) 1,2-distearoyl-*sn*-glycero-3-phosphatidylethanolamine (DSPE-PEG), and 1,2-distearoyl-*sn*-glycero-3-phosphoethanolamine-*N*-[maleimide(polyethyleneglycol)-2000] (DSPE-PEG-mal) were purchased from Xi’an ruixi Biological Technology Co. Ltd. (Xian, China). Coumarin-6 was obtained from Sigma-Aldrich Co. (St Louis, MO, USA). Sephadex G-50 was obtained from GE Healthcare Life Sciences (WI, USA). Recombinant human PDGF-BB was purchased from PEPROTECH (Rocky Hill, USA). The LXRα agonist, T0901317, was purchased from Selleckchem (Boston, USA). Cell cycle analysis kits were obtained from BD Bioscience (San Jose, CA, USA). BrdU Cell Proliferation Detection Kits were obtained from KeyGen BioTECH (Nanjing, China). Mouse aortic smooth muscle (MOVAS) cells were purchased from Honsun Biologicals (Shanghai, China). Dulbecco’s modified Eagle’s medium (DMEM) and fetal bovine serum (FBS) were purchased from HyClone (Logan, UT, USA). Anti-Rb antibodies targeting the total and phosphorylated (Ser 807/811) forms, as well as minichromosomal maintenance protein 6 (MCM6), and β-actin were purchased from Biosynthesis Biotechnology Co. Ltd. (Beijing, China). CCK-8 and western blot detection reagents were purchased from Amersham Life Science Beijing Solarbio Science and Technology Co. Ltd. (Beijing, China). All chemicals were analytical or of high-performance liquid chromatography (HPLC) grade and used without additional purification.

### Preparation of Multifunctional Liposomes

#### Preparation of Liposomes Loaded with T0901317 and Coumarin-6

Therapeutic-fluorescent liposomes were prepared by filming-rehydration method [19]. Briefly, PC, Chol, DSPE-PEG2000, and DSPE-PEG-mal were combined in a round-bottom flask containing 5 mL of chloroform/methanol (3:1) in a ratio of 4:1:0.8:0.2 g (50 mg in total) with two different weight ratios of T0901317. The mixture was dried using a rotary evaporator at room temperature to form a thin film. The film was then processed in a vacuum pump, hydrated for 2 h in 5 mL of PBS, and sonicated for 10 min at 30 °C. To optimise liposome formation, the mixture composition was assessed (Table 1). The weight ratios of T0901317 influenced the encapsulation percentage and loading efficiency. Undissolved T0901317 was removed by filtration. To obtain fluorescence images, NPs were visualised with 1% (*w*/*w*) coumarin-6.

**Table 1:** Physicochemical characteristics of synthetic NP formulations

Formulation	Size (nm)	PDI	ZP (mV)
T0901317- liposomes	155.8 ± 2.38	0.133 ± 0.05	−1.9 ± 0.84
Anti-ICAM-1-T0901317- liposomes	189.2 ± 2.74	0.173 ± 0.03	−18.1 ± 0.96

*PDI* polydispersity, *ZP* zeta potential

#### Preparation of Anti-ICAM-1 and T0901317 Liposome Conjugate

Anti-ICAM-1 antibodies were conjugated with T0901317 liposomes following previously described methods [20]. Briefly, T0901317 liposomes (10 mg) and anti-ICAM-1 (20 μg) were dissolved in PBS (pH = 7.4) and stirred overnight in a glass bottle. Conjugates were then purified using a Sephadex G-50 (1 × 20 cm) gel filtration column.

### Liposome Characterisation

The morphology and size of the anti-ICAM-1-T0901317- liposomes were measured by transmission electron microscopy (TEM; JEM-2100). Briefly, the liposome suspension was dropped on a copper grid and negatively stained with 1% phosphotungstic acid until dry. Hydrodynamic sizes, polydispersity (PDI), and zeta potential of the anti-ICAM-1-T0901317- liposomes and T0901317- liposomes were measured in aqueous solutions using a Zetasizer Nano ZS dynamic light scattering (DLS) instrument (DLS; Malvern Zetasizer 2000, Malvern, UK). UV spectrophotometry was used to determine whether liposomes were successfully bound to the target ICAM-1 antibodies. The absorption spectra were obtained using a UV-visible absorption spectrometer (UV-1601; Shimadzu; Kyoto, Japan) at wavelengths of 200–700 nm [21].

### Measurement of In Vitro Entrapment Efficiency, Loading Efficiency, and Drug Release

The exact amount of T0901317 in NPs was assessed by HPLC [22]. Briefly, methanol was used to establish a T0901317 standard curve. To remove free drug, liposomes were filtered, followed by sonication for 5 min in 2.0 mL of methanol. Drug quantity in the supernatant was determined by HPLC following centrifugation at 6000*g* for 15 min. The entrapment efficiency (EE) and loading efficiency (LE) were calculated according to the following formulae [23]:

$$ \mathrm{L}\mathrm{E} = \left(\mathrm{quality}\ \mathrm{of}\ \mathrm{drug}\ \mathrm{in}\ \mathrm{the}\ \mathrm{NPs}/\mathrm{quality}\ \mathrm{of}\ \mathrm{the}\ \mathrm{NPs}\right) \times 100\%; $$

$$ \mathrm{E}\mathrm{E} = \left(\mathrm{quality}\ \mathrm{of}\ \mathrm{drug}\ \mathrm{in}\ \mathrm{the}\ \mathrm{NPs}/\ \mathrm{total}\ \mathrm{in}\mathrm{put}\ \mathrm{quality}\ \mathrm{of}\ \mathrm{drug}\mathrm{s}\right) \times 100\%. $$

To measure drug release from targeted and nontargeted liposomes, liposomes were diluted in 9 mL of PBS containing 0.1% sodium dodecyl sulphate (SDS; pH 7.4) and incubated in a vibrating water bath at 37 °C and 130 rpm. Following incubations of 0–50 h, samples were centrifuged at 20,000*g* for 15 min and mixed with equal volumes of PBS containing 0.1% SDS. The drug content in the supernatant was then measured by HPLC, and the cumulative T0901317 release from control or anti-OPN liposomes was plotted by the release ratio versus time.

### Cell Experiments

#### Cell Culture

MOVAS cells were cultured in DMEM medium supplemented with 10% FBS and 1% penicillin-streptomycin. Cells were cultivated in culture bottles at 37 °C and 5% CO_2_. Cells were treated with PDGF-BB at the final concentration of 20 ng/mL. The culture medium was replaced daily, and cells were sub-cultured at 100% confluence.

#### Immunofluorescence Analysis

Treated cells were pre-incubated with PDGF-BB at the final concentration of 20 ng/mL for 24 h. Following digestion and centrifugation, cells were fixed with 4% paraformaldehyde and washed three times with ice-cold PBS containing 0.2% Triton X-100. Cells were then incubated for 1 h with primary anti-ICAM-1 antibodies in PBS containing 0.1% BSA 1. Cells were then washed three times in PBS and incubated for 1 h at 37 °C with FITC-conjugated secondary antibodies. Nuclei were stained with DAPI and washed three times. A confocal microscope (LSM510; Carl Zeiss; Germany) was used to analyse the expression of the target protein [24].

#### Cellular Uptake and Targeting Efficiency of Liposomes

MOVAS cells were cultured at 10^4^ cells per well in confocal dishes and incubated for 24 h with PDGF-BB at the final concentration of 20 ng/mL. The medium was then replaced with the same containing 40 μL of T0901317- liposomes and anti-ICAM-1-T0901317 liposomes (1 mg lipid/mL), respectively. After 4 h, the medium was removed and cells were fixed with 4% paraformaldehyde. Nuclei were stained with DAPI for an additional 10 min. After washing the cells three times with PBS, confocal microscopy was performed (LSM510; Carl Zeiss; Germany) using two channels for DAPI and coumarin-6 (466 nm excitation, 504 nm detection). To validate the cellular uptake and targeting efficiency of liposomes, flow cytometry (Gallios; Beckman Coulter; CA, USA) was performed on 10^4^ cells using an excitation wavelength of 466 nm.

#### Cell Proliferation Assay

Cellular proliferation was assessed using CCK-8 and BrdU Cell Proliferation Detection Kits. Cells were seeded in 96-well plates and treated with T0901317, anti-ICAM-1-T0901317-NPs, T0901317-NPs, and control liposomes for 24 h. Following incubation, medium was removed and cells were washed three times in PBS. The appropriate CCK-8 was added to each well and incubated for 4 h at 37 °C. The optical densities (OD) were determined at 450 nm using a microplate spectrophotometer.

Cell proliferation was also assessed by the number of nuclei that were DAPI stained and marked with BrdU. BrdU was used at a final concentration of 20 μmol/mL. The secondary antibody for BrdU was labelled TRITC. Images were obtained using an FV1200 microscope (Olympus Corporation; Japan).

#### Cell Cycle Analysis

Cells were cultured in six-well plates and incubated with T0901317, anti-ICAM-1-T0901317-NPs, T0901317-NPs, and blank liposomes for 24 h. Following incubation, medium was discarded and cells were washed three times in PBS. Treated cells were incubated with PDGF-BB for 24 h at a final concentration of 20 ng/mL, after which they underwent sequential incubation in trypsin, trypsin inhibitor and RNase buffer, and PI (propidium iodide) stain. Cells were incubated for 10 min on ice in the dark and assessed by flow cytometry (Gallios, Beckman Coulter; CA, USA).

#### Western Blot Analysis

Western blot analyses were performed as previously described [25]. Total protein was extracted using 100 μL of radioimmunoprecipitation assay (RIPA) buffer and 1 μL of phenylmethylsulfonyl fluoride (PMSF) and quantified using the BCA Protein Assay (Pierce Biotechnology; MA, USA). Samples were separated using sodium dodecyl sulphate-polyacrylamide gel electrophoresis (SDS-PAGE) and transferred to nitrocellulose membranes. Membranes were blocked with 5% (*w*/*v*) bovine serum albumin (BSA) at room temperature for 30 min and incubated with appropriate primary and secondary antibodies. Blots were developed using the Supersignal West Pico Chemiluminescent Substrate kit (Pierce Biotechnology; MA, USA) and analysed using ImageJ software (National Institutes of Health; USA).

### Statistical Analysis

Data were analysed using SPSS.17 software and expressed as the means ± SEM. Assessments of statistical significance were performed using ANOVA and *t* tests (paired and unpaired). A *P* value of <0.05 was considered statistically significant.

## Results and Discussion

### The Scheme of Study Design

The scheme representing the subject of the article shows that the anti-ICAM-1-T0901317 liposomes were prepared by filming-rehydration method. Some specific materials contain phospholipid, cholesterol, T0901317, DSPE-PEG-mal, coumarin-6, and anti-ICAM-1. And the anti-ICAM-1-T0901317 liposomes can inhibit PDGF-induced proliferation of vascular smooth muscle cells effectively (Fig. 1).

### Characterisation of Liposomes

**Fig. 2:**
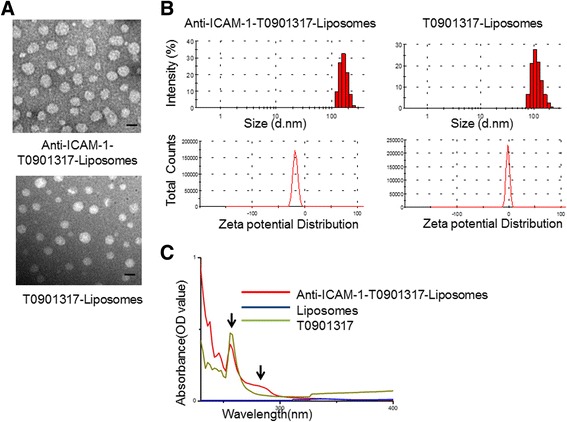
Characterisation of nanoparticles. **a** The picture above is the TEM image of the antibody T0901310 liposomes. And the photo below is the TEM image of the T0901310- liposomes. *Bar* = 100 nm. **b** Hydrodynamic diameter and zeta potentials for antibody T0901317 liposomes and T0901317 liposomes. **c** UV-visible absorption values of anti-ICAM-1-T0901317- liposomes, control liposomes, and T0901317 liposomes. And the different DLS size changes of these liposomes in different pH values are shown in Additional file 1: Figure S1

TEM assessments (Fig. 2a) revealed that the anti-ICAM-1-T0901317 liposomes were spherical and 130 nm in diameter. The hydrodynamic diameter and the zeta potential of T0901317 liposomes and anti-ICAM-1-T0901317 liposomes were determined by DLS analysis and are shown in Fig. 2b. The zeta potential of liposomes changed from neutral to negative, and a decrease in hydrodynamic diameter was observed upon treatment with liposome-linked antibodies. These changes were due to the negative charge of the antibody itself. The hydrodynamic diameter and zeta potential of anti-ICAM-1-T0901317 liposomes were 189.2 ± 2.74 nm and −18.1 ± 0.96 mV, respectively, and the hydrodynamic diameter and zeta potential of T0901317 liposomes were 155.8 ± 2.38 nm and −1.9 ± 0.84 mV, respectively. Absorption spectra of anti-ICAM-1-T0901317 liposomes at 256 and 280 nm indicated that cells contained drug-linked antibodies.

### Entrapment Efficiency, Loading Efficiency, and Drug Release

Lipid to T0901317 weight ratios of 5:1 and 10:1 were used in the evaporation process (50 mg total). The encapsulation efficiency was lower for the anti-ICAM-1-T0901317 liposomes when the ratio was 5:1, and drug loading was evaluated for all subsequent experiments. No significant differences in entrapment efficiencies were observed with antibody conjugation.

**Fig. 3:**
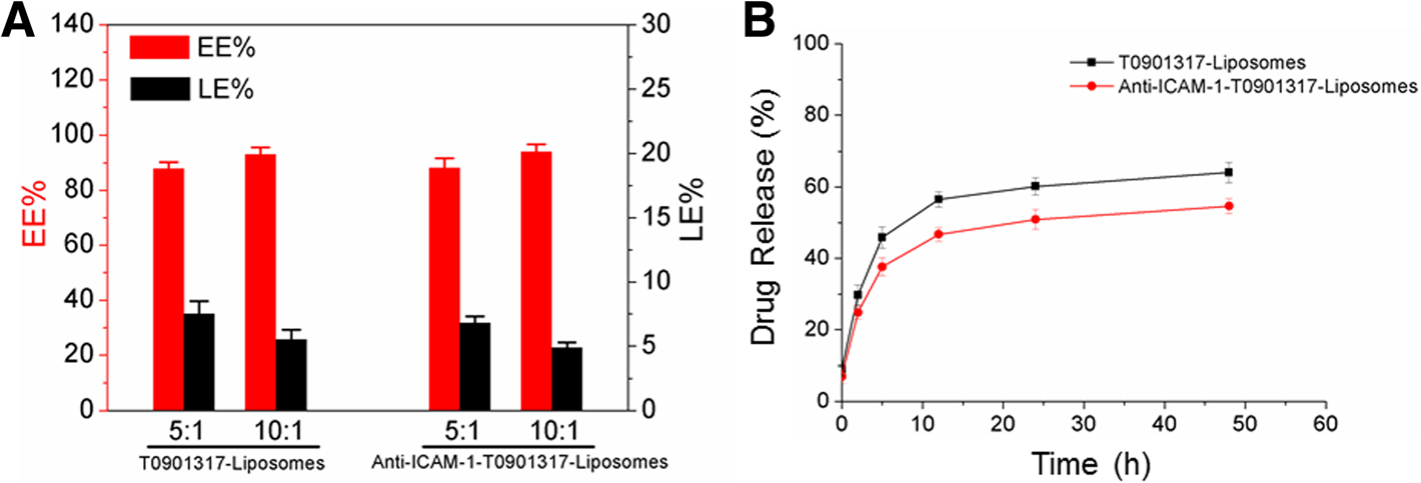
**a** Entrapment and loading efficiencies of T0901317- liposomes and anti-ICAM-1-T0901317- liposomes at two lipid to T0901317 weight ratios (5:1 and 10:1). **b** In vitro drug release of T0901317- liposomes (*squares*) and anti-ICAM-1-T0901317- liposomes (*circles*) at different time points. Values are expressed as the means ± SD (*n* = 3)

Drug release from both types of liposomes was biphasic (Fig. 3b). The differences in release were reduced over 5–50 h, which may have been dependent upon whether the encapsulated drug was inside the liposome or surface exposed [26]. Drug release from the anti-ICAM-1-T0901317 liposomes was slower than that of the T0901317- liposomes. The HPLC result for the T0901317 is shown in Additional file 2: Figure S2.

### Influence of PDGF-BB on Expression of ICAM-1 in VSMCs

**Fig. 4:**
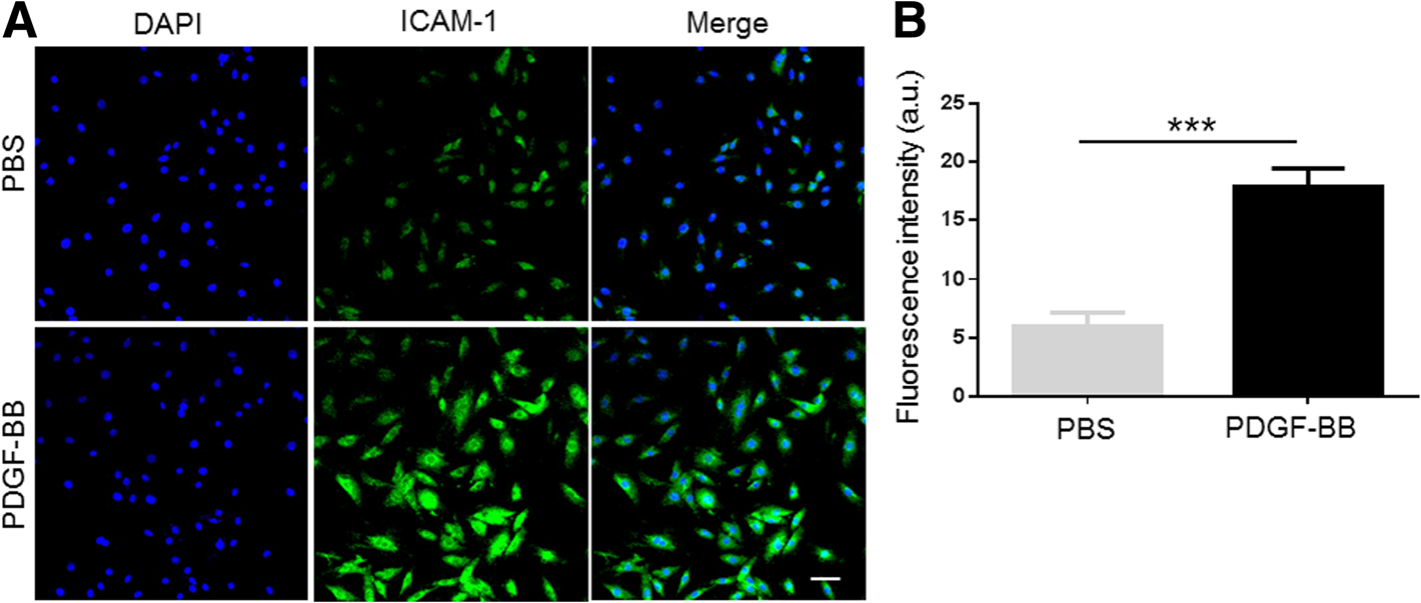
**a** Immunofluorescence images of ICAM-1 expression in MOVAS cells following treatment with platelet-derived growth factor (PDGF)-BB (20 μg/mL) for 24 h. *Left column* DAPI channel showing blue fluorescence, *middle column* FITC channel showing green fluorescence from the liposomes, *right column* DAPI and FITC channels merged. *Bars* = 50 μm. **b** Mean FITC fluorescence intensity obtained from images in **a**. Values are expressed as the means ± SD (*n* = 3). ***P < 0.01

Immunofluorescence was used to evaluate the expression of ICAM-1 in VSMCs following PDGF-BB treatment (Fig. 4a). Quantitative fluorescence analyses were performed using ImageJ software. Treatment of MOVAS with PDGF-BB stimulated ICAM-1 expression (Fig. 4b).

### Cellular Uptake and Targeting Efficiency in Liposomes

**Fig. 5:**
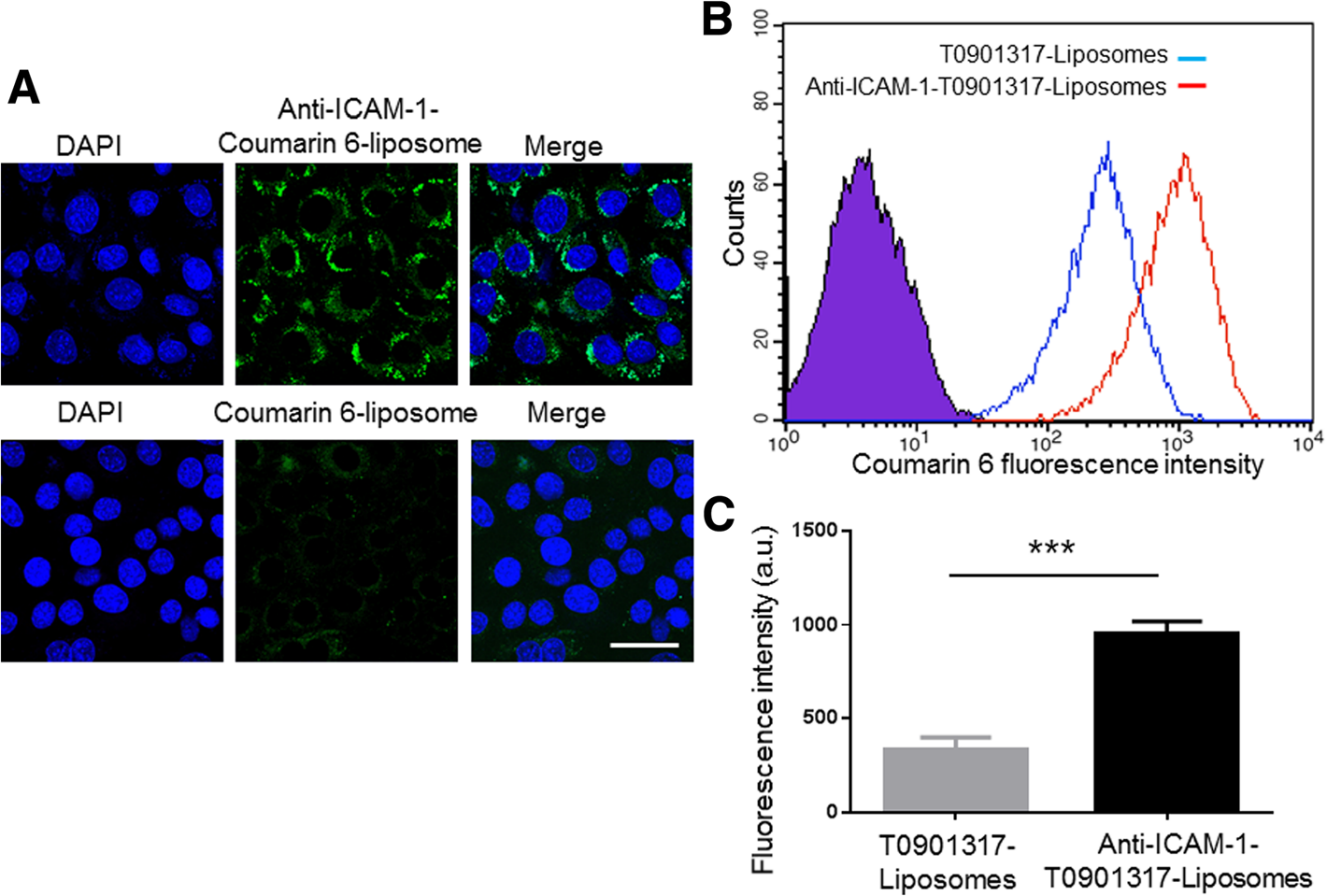
Cellular uptake and targeting efficiency of coumarin-6 in liposomes. **a** CLSM images of MOVAS cells pre-treated for 24 h with PDGF-BB (20 μg/mL), followed by incubation with anti-ICAM-1-coumarin-6 liposomes, or coumarin-6 liposomes for 4 h. *Left column* DAPI channel showing *blue* fluorescence, *middle column* coumarin-6 channel showing *green* fluorescence from the liposomes, *right column* merged channel showing DAPI and coumarin-6. *Bars* = 50 μm. **b** Flow cytometry analyses of MOVAS cells pre-treated for 24 h with PDGF-BB (20 μg/mL) followed by treatment with anti-ICAM-1-coumarin-6 liposomes or coumarin-6 liposomes for 4 h. Emitted light was collected at FL6 using a 660/20-nm band pass filter. **c** Mean coumarin-6 fluorescence intensities obtained from MOVAS cells pre-treated for 24 h with PDGF-BB (20 μg/mL) and incubated with anti-ICAM-1-coumarin-6 liposomes or coumarin-6 liposomes for 4 h. Values are expressed as the means ± SD (*n* = 3); **P < 0.01

The targeting effects of anti-ICAM-1-T0901317 liposomes on cells were assessed using coumarin-6 for detection. Following treatment of MOVAS cells with PDGF-BB (20 ng/mL) for 24 h, cells were incubated for 4 h with each of the two groups of liposomes. Cellular uptake was then evaluated by confocal laser scanning microscope (CLSM) and flow cytometry. As can be seen from Fig. 5a, the uptake of anti-ICAM-1-T0901317 liposomes was significantly higher than the uptake from the second group. Similarly, the fluorescence intensity of intracellular coumarin-6 was significantly higher in anti-ICAM-1-T0901317- liposomes, compared to the other group (Fig. 5b). The mean fluorescence intensity of intracellular anti-ICAM-1-T0901317- liposomes was ~2.8-fold higher than that of T0901317- liposomes, suggesting that anti-ICAM-1-T0901317 liposomes target MOVAS cells. In addition, the fluorescence spectra of the anti-ICAM-1-T0901317- liposomes are presented in Additional file 3: Figure S3.

### Cell Proliferation Assessments by CCK-8 Assay and BrdU Immunofluorescence

**Fig. 6:**
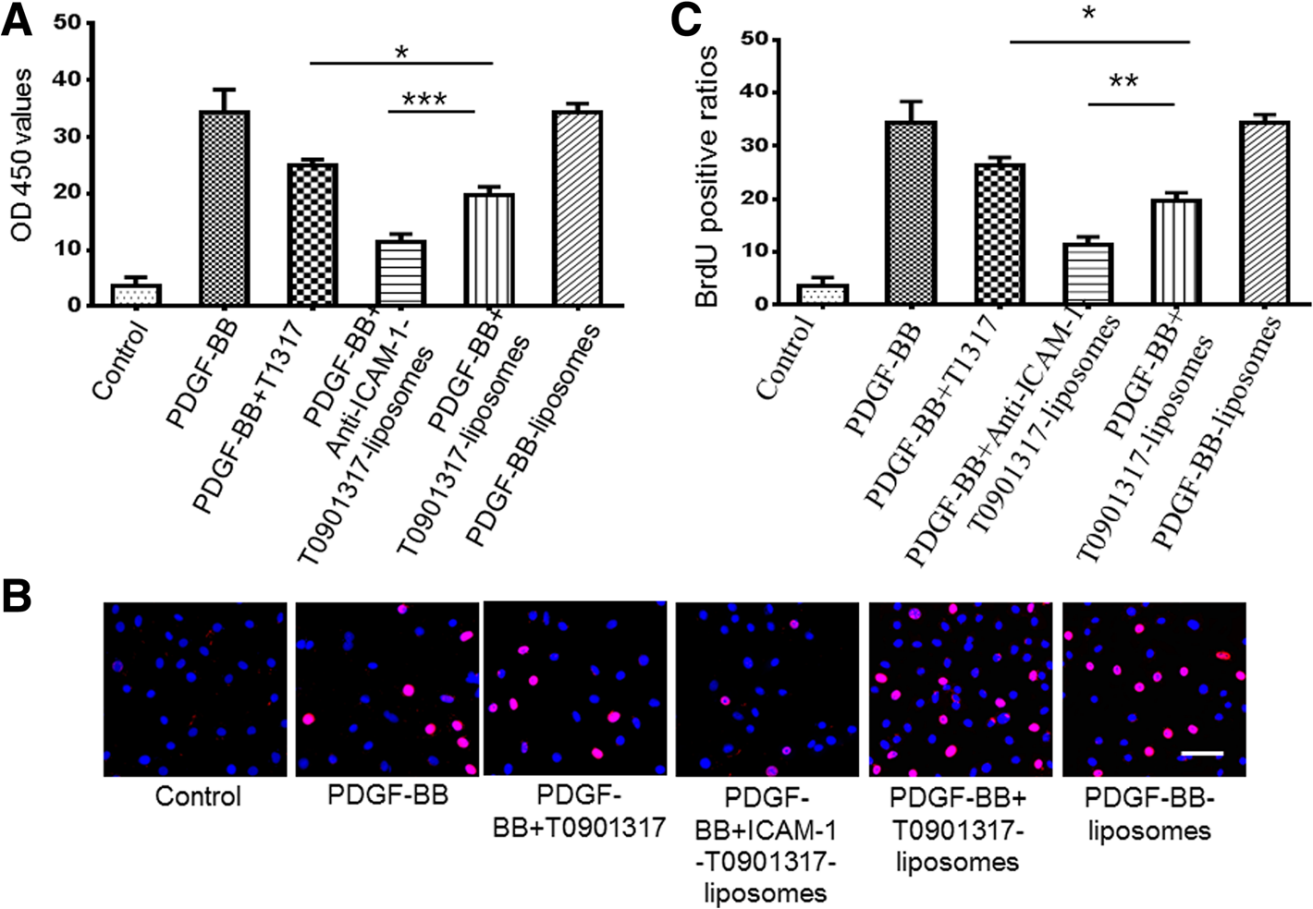
Cell proliferation assessments using the CCK-8 assay and BrdU immunofluorescence (**a**). The CCK8 proliferation assay showed that anti-ICAM-1-T0901317 liposomes prevented the proliferation of vascular smooth muscle cells (VSMCs) induced by PDGF-BB more substantially than T0901317 liposomes and T0901317. **b** Confocal images showing that administration of anti-ICAM-1-T0901317 liposomes decreased the number of BrdU+ cells following treatment with PDGF-BB (20 μg/mL) for 24 h more substantially than T0901317- liposomes or T0901317. *Bars* = 100 μm. **c** Graphs of BrdU-positive ratios. All the experiments were repeated three times and represented as the means ± SEM. *P < 0.05; **P < 0.01; ***P < 0.001

Cell proliferation was measured using the CCK-8 assay and BrdU immunofluorescence. During the CCK-8 assay, MOVAS cells were pre-treated for 24 h with PDGF-BB (20 ng/mL) followed by treatment with anti-ICAM-1-T0901317 liposomes for 24 h. The inhibitory effect of anti-ICAM-1-T0901317- liposomes on cell proliferation was subsequently assessed, and it was found that targeted liposomes were more effective than nontargeted liposomes and T0901317 molecules (Fig. 6a). The controls had no effect on cell proliferation. Cell proliferation was also assessed using BrdU staining, which revealed that anti-ICAM-1-T0901317- liposomes suppressed PDGF-BB-induced proliferation of vascular smooth muscle cells, compared to T0901317- liposomes and T0901317 alone (all *P* < 0.05). No significant difference in cell proliferation was observed between the PDGF-BB or control groups (*P* > 0.05).

### Anti-ICAM-1-T0901317 Liposomes Inhibited PDGF-BB-Induced MOVAS Cell Proliferation and G_1_ → S Phase Progression

**Fig. 7:**
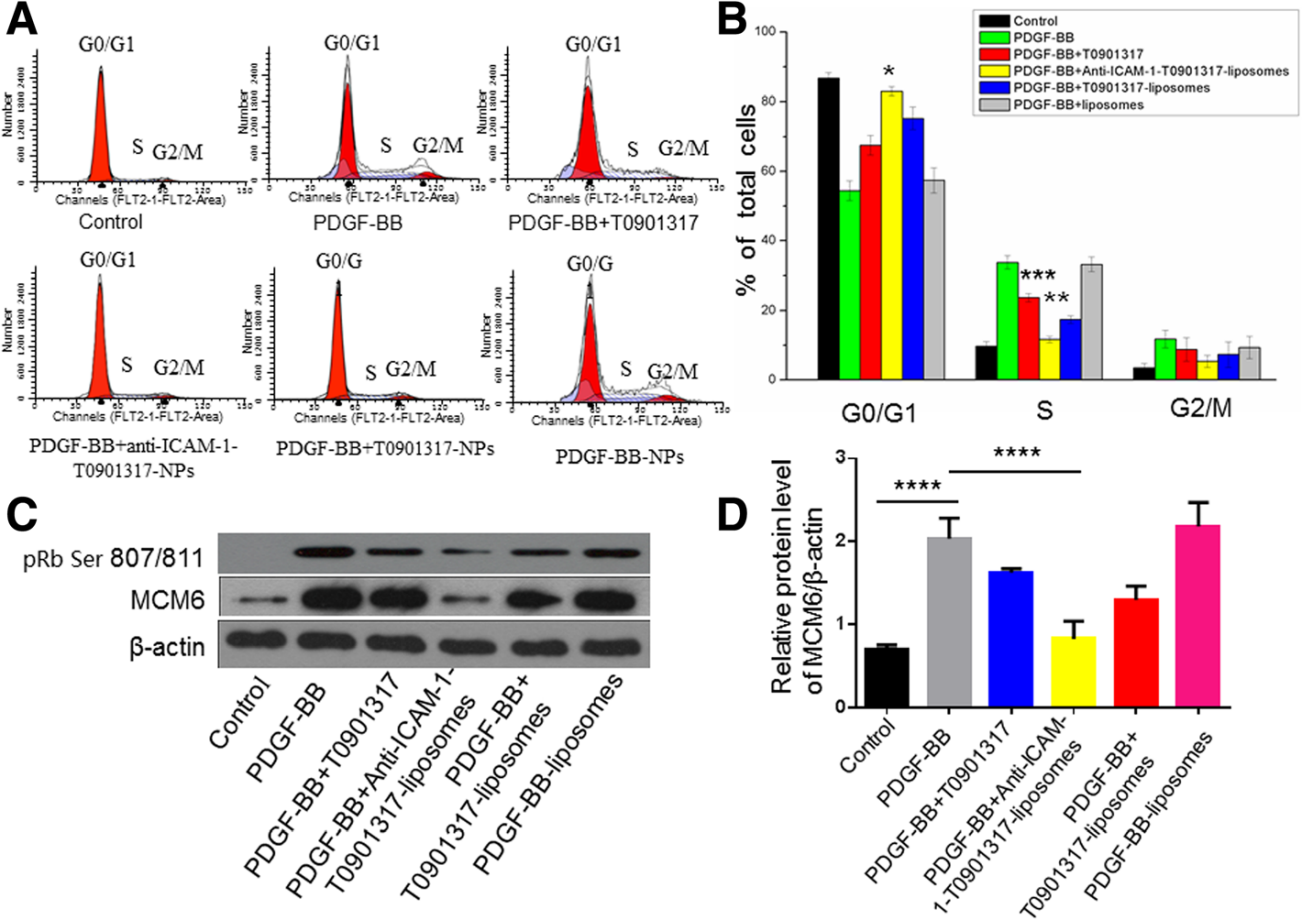
Anti-ICAM-1-T0901317 liposomes inhibit PDGF-BB-induced G1 → S phase progression by inhibiting Rb phosphorylation and MCM6 expression. Cells were pre-treated for 24 h with PDGF-BB (20 μg/mL) followed by treatment with targeted and nontargeted liposomes, T0901317, or control liposomes. **a** Cell cycle progression was assessed by flow cytometry. Anti-ICAM-1-T0901317 liposomes caused cells to remain in G1. **b** Cell cycle quantification is represented by the histogram. **c** Whole-cell proteins were assayed by western blotting. **d** Bar graphs showing the quantification of western blots. Data are expressed as a percentage of the control. Values were expressed as the means ± SD (*n* = 3). **P < 0.01

The effect of anti-ICAM-1-T0901317 liposomes on cell cycle progression was determined by flow cytometry. To analyse the effect on cell proliferation, it was necessary to analyse the effect of anti-ICAM-1-T0901317 liposomes on the cell cycle. Cells incubated with PDGF for 24 h preferentially accumulated in S phase (54.4 ± 2.8% in G0/G1 phase and 33.8 ± 2.0% in S phase; Fig. 7a). Treatment with T0901317 liposomes suppressed progression into S phase, causing an increase in the G0/G1 population (75.3 ± 3.2%). Additionally, cells incubated with anti-ICAM-1-T0901317 liposomes for 24 h primarily accumulated in G0/G1 phase (83.0 ± 1.3%). However, cells treated with control nanomaterials following incubation with PDGF-BB for 24 h did not exhibit altered cell cycles.

### Anti-ICAM-1-T0901317 Liposomes Inhibit Phosphorylation of Rb and Expression of MCM6

T0901317 inhibits cell cycle progression by inhibiting the expression of Rb phosphorylated at Ser807/811, which is necessary for G_1_ → S progression. Moreover, Rb phosphorylation leads to increased MCM6 expression [17]. As can be seen in Fig. 7, anti-ICAM-1-T0901317 liposomes inhibited Rb phosphorylation and MCM6 expression more than other groups. No significant difference in protein expression was observed when MOVAS cells were pre-treated for 24 h with PDGF-BB (20 ng/mL) followed by targeted nanoparticles treatment with empty controls (Fig. 7).

## Conclusions

VSMC proliferation plays an important role in the development of atherosclerosis, for which PDGF is a key mediator [27]. The LXR agonist, T0901317, regulated lipid accumulation in VSMCs and inhibited smooth muscle cell proliferation by preventing the phosphorylation of Rb. Thus, T0901317 may play a protective role in preventing atherosclerotic plaque formation [17, 28]. ICAM-1, which is highly expressed following PDGF-BB treatment, was chosen as the target of sustained-release liposomes containing T0901317. The goal was to inhibit smooth muscle cell proliferation [29, 30]. Liposomes were used since they have many advantages, including targeted diagnoses and therapies, sustained drug release, and suitable hydrophobicities. The physicochemical characteristics of liposomes were determined by assessing their sizes, morphologies, zeta potentials, stabilities, entrapment efficiencies, loading efficiencies, and drug releases. Notably, the therapeutic effect of liposomes on an in vitro model of atherosclerosis has been validated. The data from this study show that targeted diagnosis and treatment of atherosclerosis is possible.

## Additional files

Additional file 1: **Figure S1.** DLS size of these liposomes in different pH values. As can be seen, the liposomes are relatively stable in different pH environments. Smaller size of particle may be caused by differences in solution charge. (TIF 75 kb)

Additional file 2: **Figure S2.** This figure shows the results of the T0901317 in HPLC. The peak time of the T0901317 in HPLC was about 5.8 min. The sample is detected for 36 min. (TIF 69 kb)

Additional file 3: **Figure S3.** The fluorescence spectra of the anti-ICAM-1-T0901317 liposomes were presented. The optimal absorption wavelength of liposomes is 518 nm. It can be seen that it is consistent with the fluorescent properties of the coumarin-6. (TIF 130 kb)
