# Supplementary figures and images for: ICAM-1-Targeted Liposomes Loaded with Liver X Receptor Agonists Suppress PDGF-Induced Proliferation of Vascular Smooth Muscle Cells
Source: Nanoscale Res Lett. 2017 May 3;12:322. doi: 10.1186/s11671-017-2097-6 (PMC5415450; doi:10.1186/s11671-017-2097-6)

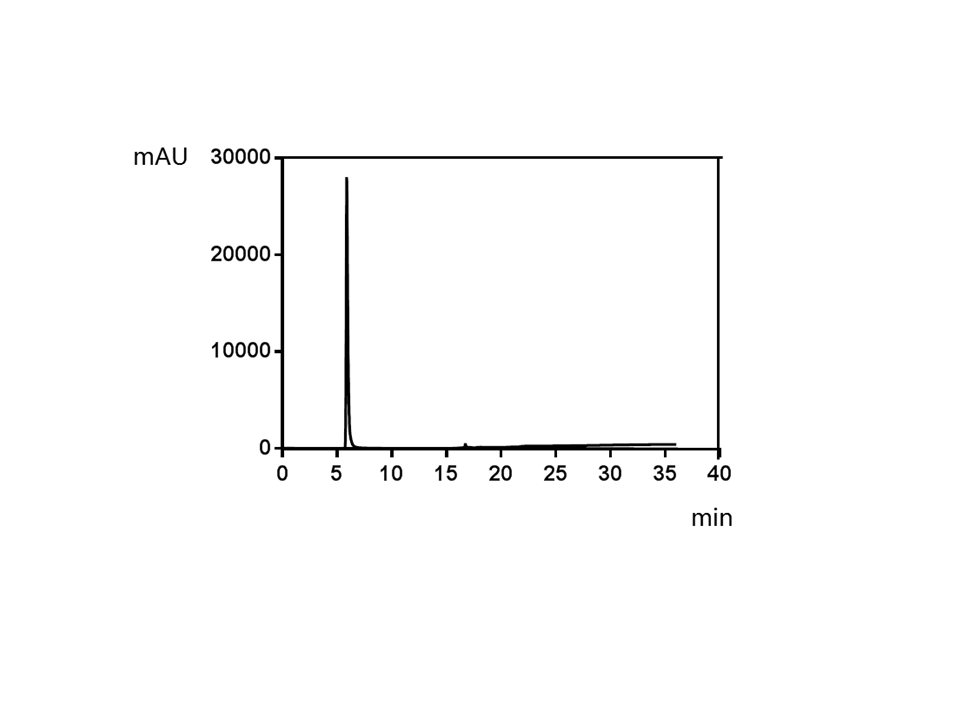

Supplement: Supplementary file 1 — Figure S1. DLS size of these liposomes in different pH values. As can be seen, the liposomes are relatively stable in different pH environments. Smaller size of particle may be caused by differences in solution charge. (TIF 75 kb) [file 11671_2017_2097_MOESM1_ESM.tif]

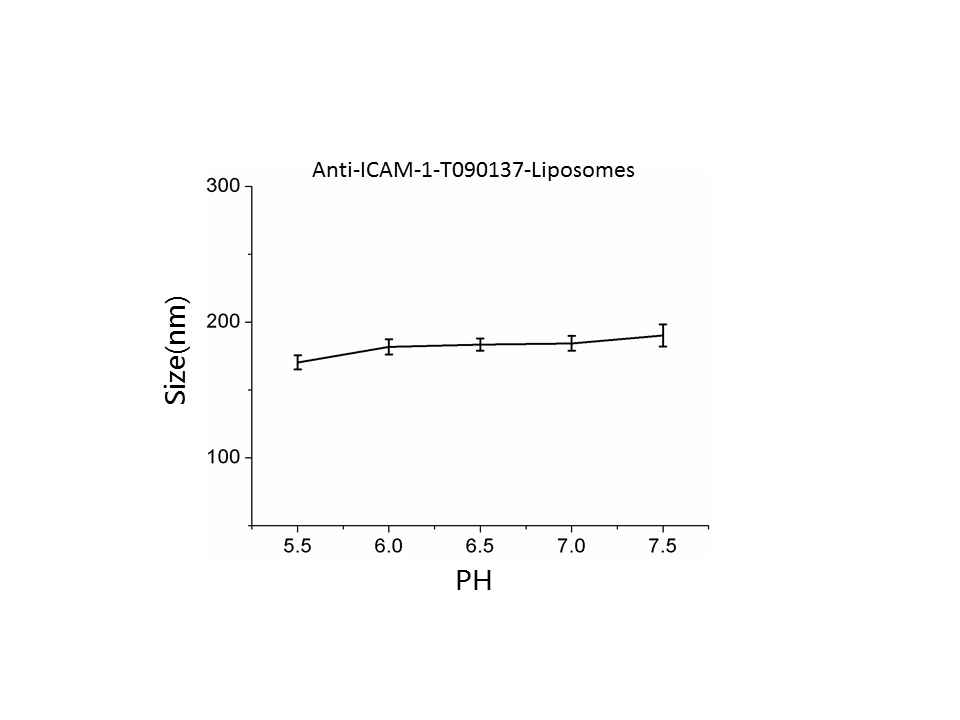

Supplement: Supplementary file 2 — Figure S2. This figure shows the results of the T0901317 in HPLC. The peak time of the T0901317 in HPLC was about 5.8 min. The sample is detected for 36 min. (TIF 69 kb) [file 11671_2017_2097_MOESM2_ESM.tif]

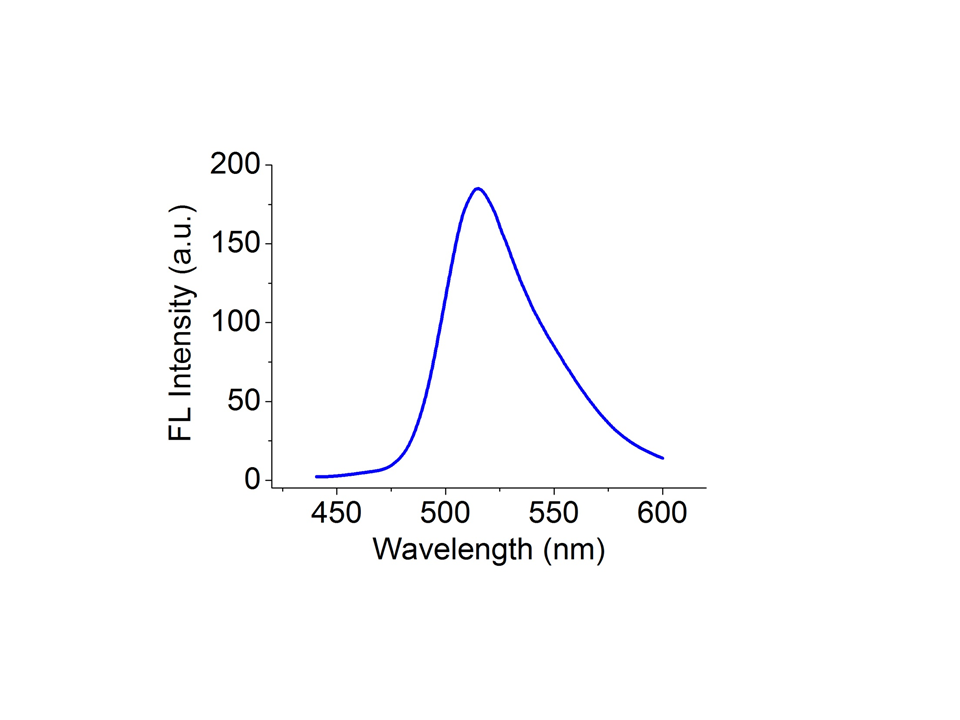

Supplement: Supplementary file 3 — Figure S3. The fluorescence spectra of the anti-ICAM-1-T0901317 liposomes were presented. The optimal absorption wavelength of liposomes is 518 nm. It can be seen that it is consistent with the fluorescent properties of the coumarin-6. (TIF 130 kb) [file 11671_2017_2097_MOESM3_ESM.tif]
